# Neoadjuvant immunotherapy across cancers: meeting report from the Immunotherapy Bridge—December 1st–2nd, 2021

**DOI:** 10.1186/s12967-022-03472-x

**Published:** 2022-06-15

**Authors:** Elizabeth M. Burton, Rodabe N. Amaria, Tina Cascone, Myriam Chalabi, Neil D. Gross, Elizabeth A. Mittendorf, Richard A. Scolyer, Padmanee Sharma, Paolo A. Ascierto

**Affiliations:** 1 Department of Genomic Medicine, The University of Texas MD Anderson Cancer Center, Houston, TX USA; 2 Department of Melanoma Medical Oncology, University of Texas MD Anderson Cancer Center, Houston, TX USA; 3 Department of Thoracic/Head and Neck Medical Oncology, University of Texas MD Anderson Cancer Center, Houston, TX USA; 4 Gastrointestinal Oncology, Netherlands Cancer Institute, Amsterdam, the Netherlands; 5 Department of Head and Neck Surgery, University of Texas MD Anderson Cancer Center, Houston, TX USA; 6 Division of Breast Surgery, Department of Surgery, Brigham and Women’s Hospital/Breast Oncology Program, Dana-Farber Brigham Cancer Center, Boston, MA USA; 7 Melanoma Institute of Australia, The University of Sydney/Faculty of Medicine and Health, The University of Sydney/Tissue Pathology and Diagnostic Oncology, Royal Prince Alfred Hospital and NSW Health Pathology/Charles Perkins Centre, The University of Sydney, Sydney, NSW Australia; 8 Immunotherapy Platform, Department of Immunology/Department of Genitourinary Medical Oncology, Division of Cancer Medicine, University of Texas MD Anderson Cancer Center, Houston, TX USA; 9 Department of Melanoma, Cancer Immunotherapy and Innovative Therapy, Istituto Nazionale Tumori IRCCS “Fondazione G. Pascale”, Naples, Italy

**Keywords:** Immunotherapy, Checkpoint inhibitors, PD-1, CTLA-4, Neoadjuvant, Pathological response

## Abstract

After the success of immunotherapy in the treatment of advanced metastatic cancer, further evaluation in earlier settings, including high-risk, surgically-resectable disease is underway. Potential benefits of a neoadjuvant immunotherapeutic approach include presurgical tumor shrinkage, reduced surgical morbidity, early eradication of micrometastases and prevention of distant disease, and greater antigen-specific T cell response. For some cancers, pathologic response has been established as a surrogate measure for long-term outcomes, therefore offering the ability for early and objective assessment of treatment efficacy and the potential to inform and personalize adjuvant treatment clinical decision-making. Leveraging the neoadjuvant treatment setting offers the ability to deeply interrogate longitudinal tissue in order to gain translatable, pan-malignancy insights into response and mechanisms of resistance to immunotherapy. Neoadjuvant immunotherapy across cancers was a focus of discussion at the virtual Immunotherapy Bridge meeting (December 1–2, 2021). Clinical, biomarker, and pathologic insights from prostate, breast, colon, and non-small-cell lung cancers, melanoma and non-melanoma skin cancers were discussed and are summarized in this report.

## Introduction

The development of immunotherapy has revolutionized treatment of metastatic cancer and improved survival for patients across many types of cancer. After demonstrating marked success in the treatment of advanced malignancies, further evaluation of immunotherapy in earlier settings, including high-risk, surgically-resectable disease has been under investigation. Earlier administration of therapies shown to be effective in a metastatic setting may offer further benefits and neoadjuvant therapy has an established role in several cancers. Notable benefits of a neoadjuvant approach include reduced surgical morbidity through tumor shrinkage, and the opportunity for early eradication of micrometastases and prevention of distant disease. Preclinical data suggest neoadjuvant checkpoint inhibition is superior to adjuvant checkpoint inhibition, with increased eradication of distant metastases, improved survival and greater antigen-specific T cell response following primary tumor resection in a murine breast cancer model [1]. For some cancers, pathologic response has been established as a surrogate measure for long-term outcomes, therefore offering the ability for early and objective assessment of treatment efficacy and the potential to inform and personalize adjuvant treatment clinical decision-making [2–4] Leveraging the neoadjuvant treatment setting offers the ability to deeply interrogate longitudinal tissue in order to gain translatable, pan-malignancy insights into response and mechanisms of resistance to immunotherapy.

Neoadjuvant immunotherapy across cancers was a focus of discussion at the virtual Immunotherapy Bridge meeting (December 1–2, 2021). Clinical, biomarker, and pathologic insights from prostate, breast, colon, and non-small-cell lung cancers (NSCLC), melanoma and non-melanoma skin cancers were discussed and are summarized in this report.

## From the clinic to the laboratory: investigating mechanism of resistance and response to immune checkpoint therapy

Large numbers of clinical immunotherapy trials are ongoing, which include patients who are polymorphic and with heterogeneous disease. These studies can generate hypotheses in a realistic clinical setting which can then be tested in the laboratory in appropriate models in a reverse translational approach. However, clinical trial design has had to be rethought to obtain appropriate clinical samples for laboratory studies. Presurgical or tissue-based phase Ia or IIa neoadjuvant trials can allow for evaluation of clinical signals in new tumor types, biomarker analysis and mechanistic insights, such as through immune monitoring and gene expression profiling in pre- and post-treatment tumor samples. The first of these neoadjuvant trials was in 2006, prior to any US Food and Drug Administration approvals, and assessed anti-cytotoxic T-lymphocyte-associated antigen (CTLA)-4 in 12 patients with localized bladder cancer [5]. This showed a clinical signal for safety and efficacy, with three patients achieving a pathological complete response (pCR). Increased expression of inducible costimulator (ICOS), a T-cell-specific molecule that belongs to the CD28/CTLA-4/B7 immunoglobulin superfamily, was observed in tumors treated with anti-CTLA-4 antibody. In another neoadjuvant trial in 24 patients with localized urothelial cancer, durvalumab, an anti-programmed death-ligand (PD-L)1 antibody, combined with anti-CTLA-4, resulted in a 37.5% pCR rate [6]. Increased density of tertiary lymphoid structures and increased expression of ICOS + CD4 T cells was associated with improved response. In a murine melanoma model, antitumor T-cell responses to anti-CTLA-4 were significantly reduced in ICOS-deficient versus ICOS-sufficient animals [7]. These data suggest the ICOS/ICOS ligand pathway is necessary for optimal antitumor response to anti-CTLA-4, meaning it may be a therapeutic target.

In general, higher tumor mutational burden (TMB) is associated with better response to immune checkpoint blockade. Prostate cancer is typically poorly immunogenic. In patients with metastatic castration-resistant prostate cancer, high intratumoral CD8 T cell density and an interferon (IFN)-γ response gene signature and/or antigen-specific T cell responses were associated with a favorable response to ipilimumab [8]. Mutations in prostate cancer were recognized by T cells, although the extent to which T cells infiltrate into prostate tumors is unclear. In a neoadjuvant trial of ipilimumab plus androgen deprivation therapy in patients with localized prostate cancer, an increase in CD4 and CD8 T cells, including PD-1 + and ICOS + subsets, were observed after ipilimumab [9]. There were significantly higher frequencies of CD4, CD8, and ICOS + T cells in post-treatment tumors and significant increases in tumor-infiltrating immune cells, including CD4 + , CD8 + , ICOS + , CD45RO + , granzyme-B + , and CD68 + cells. Ipilimumab resulted in increased PD-L1 and V-domain Ig suppressor of T cell activation (VISTA) expression in post-treatment prostate tumors, both of which are potent inhibitors of human T cell responses. PD-L1 + and VISTA + macrophages (CD68 +) manifest an M2-phenotype and suppress T cell function. These data suggest that an increase in immune cell infiltration may be insufficient to generate antitumor responses and blockade of other immune checkpoints such as PD-L1 may be needed to provide clinical benefit for patients with prostate cancer.

Based on these data, the combination of nivolumab 1 mg/kg plus ipilimumab 3 mg/kg were evaluated in the CheckMate 650 trial in patients with metastatic castration-resistant prostate cancer [10]. Objective response rates (ORR) were 25% in a pre-chemotherapy cohort and 10% in a post-chemotherapy cohort; four patients, two in each cohort, had complete responses. However, increased toxicity with the combination has resulted in the optimal dosing regimen being further considered.

There are multiple immune checkpoints, which are dynamic in their expression and so should be evaluated in both pre- and on-treatment tumor samples to guide clinical-decision making. Neoadjuvant trials provide clinical benefit for patients with localized disease, as well as offering mechanistic insights that can be used to develop combination therapies.

## Breast cancer

Immunotherapy with PD-1/PD-L1 blockade was first approved for patients with metastatic triple-negative breast cancer (TNBC) based on the IMpassion 130 trial of atezolizumab and KEYNOTE-355 trial of pembrolizumab [11, 12]. These findings raised the questions of whether the addition of immunotherapy to chemotherapy would be more beneficial at an earlier disease stage, whether a potential improved pCR rate is correlated with improved survival, and whether or not immunotherapy before surgery is safe.

Several neoadjuvant trials in breast cancer have explored these questions. I-SPY2 was an open-label, multicenter phase II trial in which patients with high-risk stage II/III breast cancer were adaptively randomized to neoadjuvant chemotherapy with or without pembrolizumab [13]. The addition of pembrolizumab improved pCR rates versus chemotherapy in ERBB2-negative, hormone receptor-positive/ERBB2-negative, and triple-negative cohorts. In the larger phase 3 KEYNOTE-522 trial, 1174 patients with previously untreated stage II-III TNBC were randomized to neoadjuvant chemotherapy (paclitaxel and carboplatin followed by doxorubicin or epirubicin with cyclophosphamide) with either pembrolizumab or placebo [14]. After surgery, patients received adjuvant pembrolizumab or placebo for up to 27 weeks. At the first interim analysis, the pCR rate was 13.6% higher in the pembrolizumab arm (65% vs 51%; p < 0.001), with greater benefit seen in patients with clinically node-positive disease. Patients also benefited from the addition of pembrolizumab irrespective of whether their disease was PD-L1 positive or negative, which contrasts with data in the metastatic setting where benefit was limited to PD-L1 positive patients. After a median follow-up of 15.5 months, event-free survival (EFS) was higher in the pembrolizumab arm but did not reach the prespecified p-value boundary for significance. In subsequent analysis, 3-year EFS rate was significantly improved with pembrolizumab versus placebo (84.5% vs 76.8% p = 0.0003) [15]. Patients with a pCR had good survival outcomes irrespective of whether they received pembrolizumab, whereas patients without pCR had better outcomes with pembrolizumab than placebo, suggesting an additional benefit of immunotherapy in these patients. Treatment-related adverse events of grade ≥ 3 were generally similar with and without pembrolizumab in the neoadjuvant phase, although treatment discontinuations were higher. The most frequent immune-related adverse events, 10–15% of which can be permanent, were thyroid toxicities. On the basis of this trial, pembrolizumab was approved in the US for neoadjuvant use in this patient population.

In the phase III IMpassion 031 trial, 333 patients with previously untreated stage II-III TNBC were randomized to neoadjuvant chemotherapy plus atezolizumab or placebo [16]. The addition of atezolizumab to chemotherapy resulted in a significant improvement in pathological response, with pCR being achieved by 58% of patients in the atezolizumab arm versus 41% in the placebo arm (p = 0.0044). As in KEYNOTE-522, most benefit was observed in patients with node-positive disease and benefit was seen in patients regardless of PD-L1 status.

In contrast to these two studies, pCR rate was not significantly improved by the addition of neoadjuvant atezolizumab to carboplatin and nab-paclitaxel in the NeoTrip trial of 280 patients with TNBC (43.5% vs 40.8% with placebo) [17]. Unlike KEYNOTE-522 or Impassion 031, patients only received anthracycline-based therapy post-surgery, with a neoadjuvant regimen of carboplatin and nab-paclitaxel. These data called into question the optimal chemotherapy backbone to pair with immunotherapy in the preoperative setting.

In the phase II GeparNuevo trial, 174 patients with TNBC were randomized to durvalumab or placebo for 2 weeks before the addition of nab-paclitaxel followed by epirubicin plus cyclophosphamide [18]. The trial was subsequently amended so that the initial durvalumab monotherapy ‘window’ phase was removed. Overall, the pCR rate with durvalumab was 53.4% versus 44.2% with placebo, which did not meet the prespecified p-value for significance. However, a significant benefit was seen with durvalumab in the window cohort (n = 117), with a pCR of 61.0% versus 41.4%, with placebo (p = 0.035). After a median follow-up of 42.2 months, 3-year invasive disease-free survival (DFS) was 84.9% with durvalumab vs 76.9% with placebo (hazard ratio [HR] 0.54, p = 0.0559); this was achieved event though durvalumab was only administered as neoadjuvant and not adjuvant therapy [19].

**Fig. 1:**
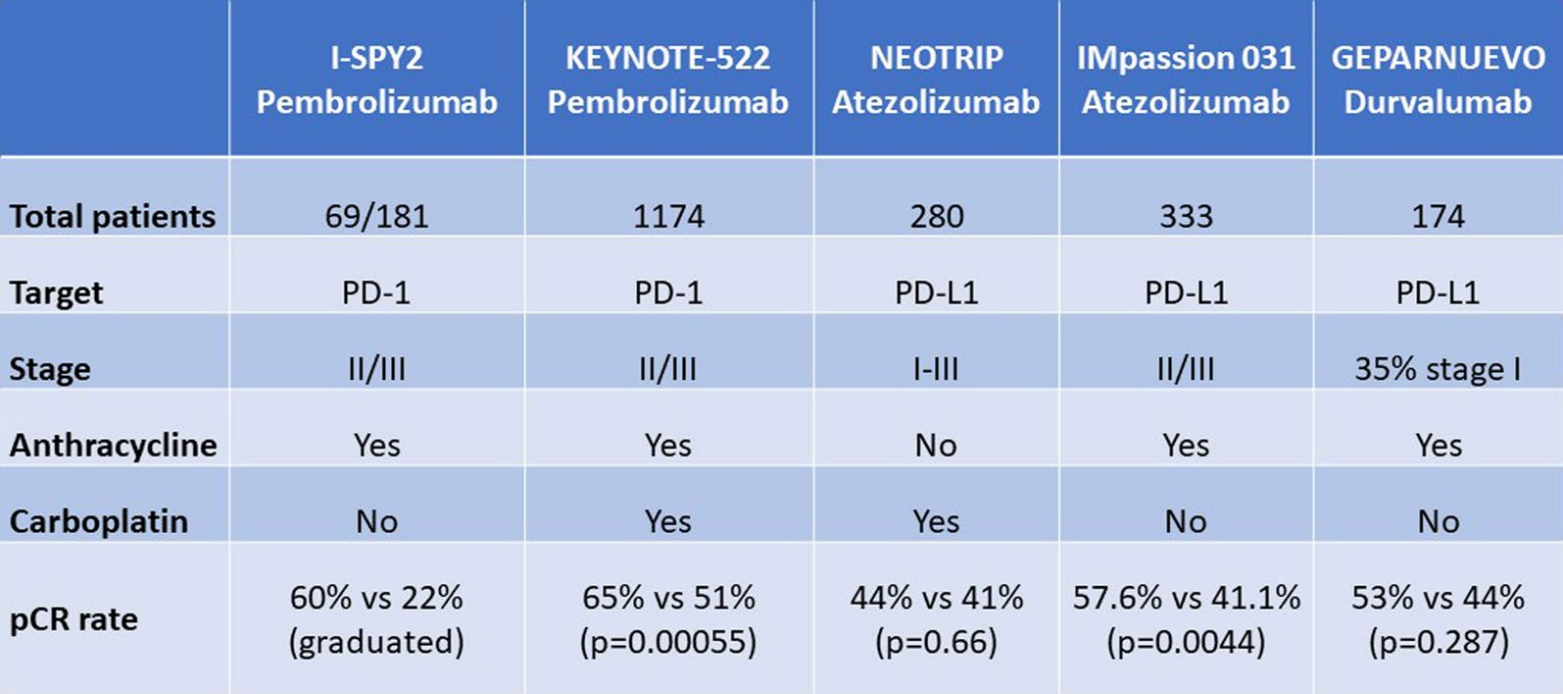
Summary Neoadjuvant Immunotherapy in Breast Cancer Studies

Overall, the KEYNOTE-522 and IMPassion 031 studies provide consistent and complementary results that are supportive of the role of neoadjuvant immunotherapy, with both studies showing an increase in pCR and benefit observed regardless of PD-L1 status. The role of adjuvant immunotherapy in patients with a pCR remains unclear. The optimal chemotherapy regimen is also still be identified. Optimal patient selection is also important; 35–50% of patients will obtain pCR with chemotherapy alone so there is a need to identify which patients might be able to avoid immunotherapy and its associated toxicities. Biomarkers of response and toxicity are also required for those patients who might benefit from immunotherapy (Fig. 1).

## Colon cancer

Immune checkpoint inhibition as monotherapy or in combination in metastatic colorectal cancer (CRC) has been associated with response rates of 30–70% in microsatellite-instability-high (MSI-H) or mismatch-repair-deficient (dMMR) tumors. However, response rates in mismatch-repair-proficient tumors (pMMR) are low. MMR deficient tumors account for only around 4% of all metastatic CRC, and 15% of early-stage CRC, although this still accounts for large numbers of patients.

Neoadjuvant immunotherapy may have higher efficacy in early-stage colon cancers. In the exploratory NICHE study, chemotherapy-naïve patients with dMMR or pMMR tumors received a single dose of ipilimumab and two doses of nivolumab before surgery [20]. In preliminary analyses of 40 patients, treatment was well-tolerated and all patients underwent surgery without delays. Over 80% of dMMR patients had stage III disease. Pathological response was observed in all 20 dMMR tumors, with 12 pCRs and 19 major pathological responses (MPRs), i.e., residual viable tumor of ≤ 10%. In pMMR tumors, 4 of 15 patients (27%) had a pathological response, with one partial response and three MPRs. Eleven patients were classed as non-responders, although four of these had tumor regression of over 10%. Of interest, these patients had a similar immune activation of the tumor microenvironment (TME) as dMMR responders. CD8 + PD-1 + T cell infiltration was found to be predictive of response in pMMR tumors.

Underestimation of pathological response using computed tomography (CT) scans was observed in several patients in the NICHE trial. Inflammation, acellular mucin and fibrosis accounted for the discordance between radiologic and pathologic responses. Potentially, the use of circulating tumor DNA and/or endoscopy may help in identifying patients who could avoid surgery in an organ-sparing approach.

The preliminary data from the NICHE study compare favorably with standard of care neoadjuvant chemotherapy in dMMR/MSI colon cancers. In the FOxTROT trial of 1053 patients with CRC, 95% of dMMR/MSI tumors had no response to chemotherapy [21]. However, immunotherapeutic responses need to be improved for pMMR patients, potentially through the use of biomarkers such as CD8 + /PD1 + T cells to select patients.

## Non-small-cell lung cancer

MPR is predictive of long-term survival benefit in neoadjuvant chemotherapy-treated patients with NSCLC [22–25] and has been implemented as candidate surrogate endpoint for evaluating novel chemotherapies and immunotherapy response, given the rare frequency of pCR following neoadjuvant chemotherapy in NSCLC (median 4%) [2].

The rationale for neoadjuvant immune checkpoint inhibition is provided by several lines of evidence. Preclinical data demonstrated that tumor PD-L1 expression facilitates the development and spread of metastasis in murine models of NSCLC [26]. In these models, genetic and pharmacologic targeting of PD-L1 on cancer cells suppresses metastasis and PD-L1 inhibition reverses the CD8 + T cell dysfunction. These results are relevant in supporting the administration of immunotherapy in the early-stage setting, given the propensity of early NSCLC to relapse with distant metastases. In early disease, an intact immune system and minimal clonal resistance also suggest an ideal setting for optimal response to immunotherapy and to reduce risk of disease recurrence.

Several trials have indicated that neoadjuvant immunotherapy is safe and effective in patients with NSCLC. Initial studies showed that neoadjuvant nivolumab was well tolerated and induced a MPR in 45% of resected tumors [27]. Neoadjuvant atezolizumab in the LCMC3 study induced MPR in 21% of patients with a pCR rate of 7% [28]. In the phase II NEOSTAR trial, 44 patients with operable NSCLC were randomized to neoadjuvant nivolumab monotherapy or nivolumab in combination with ipilimumab with MPR rate as primary endpoint [29]. The combination arm met the trial prespecified MPR efficacy boundary to be considered promising for further testing, with a 38% MPR rate versus 22% in the nivolumab arm. Combined PD-1/CTLA-4 blockade also enhanced tumor immune infiltration and immunological memory, with increased effector, tissue-resident memory and effector memory T cells in resected tumors compared with PD-1 monotherapy. Pretreatment tumor PD-L1 was associated with response to neoadjuvant immune checkpoint inhibition and increased abundance of gut *Ruminococcus* and *Akkermansia* spp. was associated with MPR to combination therapy and with favourable T cell receptor clonality at surgery [29]. Interestingly, patients treated with neoadjuvant immunotherapy demonstrated the apparent radiological progression of the disease in lymph nodes post-therapy; however, upon pathological evaluation these lymph nodes had de novo non-caseating granulomas and were devoid of cancer; this phenomenon has been described as ‘nodal immune flare’ (NIF) [30]. NIF was associated with an inflamed nodal immune microenvironment with enrichment of antigen-presenting cells, cytotoxic and T helper 1 cell markers and immune response pathways, and with fecal abundance of *Coriobacteriaceae* [30]. The NIF phenomenon should be carefully distinguished from true nodal disease progression to ensure appropriate curative-intent surgical approach.

Other studies have reported that immune checkpoint blockade combined with chemotherapy or radiation has clinical activity in patients with resectable NSCLC. The addition of neoadjuvant nivolumab, atezolizumab or durvalumab to neoadjuvant chemotherapy have all shown clinical promise in small-scale phase II studies, with encouraging MPR and pCR rates and overall good tolerability [31–33]. Neoadjuvant durvalumab combined with stereotactic body radiotherapy induced greater MPR rates compared with durvalumab monotherapy and was well tolerated [34].

Importantly, across all these studies, neoadjuvant immunotherapy did not negatively impact surgery. Delays to surgery were infrequent and resectability rates ranged from 81 to 96%, confirming that planned surgery after neoadjuvant immunotherapy is overall feasible.

In the first phase III trial of neoadjuvant immunotherapy to report, the CheckMate-816 trial, patients with resectable NSCLC were randomized to chemotherapy with or without nivolumab [35]. In the intention-to-treat population, the addition of nivolumab to platinum-based hemotherapy increased the pCR rate, assessed in both resected primary lung tumor and sampled lymph nodes, to 24% compared with 2.2% in the chemotherapy arm (p < 0.0001). The MPR rate was 37% with the nivolumab combination versus 9% with chemotherapy alone. The pCR rate was higher with neoadjuvant nivolumab plus chemotherapy as compared with chemotherapy alone across key subgroups, including disease stage, histology, tumor PD-L1 expression and TMB [35]. Definitive surgery occurred in 83% of patients treated with nivolumab plus chemotherapy and in 75% of patients treated with chemotherapy alone [36].

In conclusion, neoadjuvant immunotherapy is safe, feasible and active in patients with resectable NSCLC. The combination of nivolumab and ipilimumab induces higher MPR rates than historical controls of neoadjuvant chemotherapy and, in an exploratory analysis, than nivolumab monotherapy, and is also associated with greater tumor immune infiltrate and immunological memory than PD-1 monotherapy. CheckMate-816 is the first phase III trial to show a pCR benefit with neoadjuvant nivolumab plus chemotherapy in resectable NSCLC, with no major impact on surgery feasibility. Preclinical and translational studies are needed to identify biomarkers of response and mechanisms of resistance to these therapies, in order to develop more effective combinatorial strategies for patients with resectable NSCLC.

## Melanoma

In patients with clinical stage III melanoma, survival outcomes have historically been disappointing. Although adjuvant studies of immune- and targeted therapy have shown improved outcomes, it should be noted that these studies have screening failure rates of 15–25% due to rapid progression of disease after surgery in high-risk stage III patients. It can be postulated that patients with an aggressive disease phenotype may benefit more from a neoadjuvant approach.

In an International Neoadjuvant Melanoma Consortium (INMC) pooled analysis of six immunotherapy or BRAF targeted therapy clinical trials, pCR correlated with improved recurrence-free survival (RFS) (2-year RFS 89% vs. 50% in patients with or without pCR, p < 0.001) and overall survival (OS) (95% vs. 83%, p = 0.027) [4]. In patients with pCR, near pCR or partial pathological response with immunotherapy, very few relapses were seen, with a 2-year RFS of 96%. However, only a complete response to targeted therapy was associated with RFS, any pathological response (including a partial response) was associated with improved RFS in patients receiving neoadjuvant immunotherapy.

Several studies have investigated the effects of neoadjuvant checkpoint inhibition in melanoma. In a study of 27 patients receiving single-dose neoadjuvant plus adjuvant pembrolizumab, eight had a pCR or MPR [37]. All eight patients remained disease-free at a median follow-up of 18 months. Baseline proliferation of CD8 T cells was associated with clinical benefit and transcriptional analysis revealed a pretreatment immune signature predictive of response. In a randomized phase II study of neoadjuvant nivolumab versus ipilimumab combined with nivolumab in 23 patients with high-risk resectable melanoma, responses in the monotherapy arm were modest (ORR 25%, pCR 25%) [38]. Two patients progressed to metastatic disease during treatment and one shortly after surgery. Two patients had local recurrence after adjuvant therapy. However, the regimen was well tolerated with only 8% of patients with grade 3–4 treatment-related adverse events. In comparisons, the combination arm had high response rates (ORR 73%, pCR 45%). However, the regimen was associated with significant toxicity, with 73% of patients having grade 3–4 toxicities. Responders tended to have a higher TMB, higher PD-L1 expression and higher CD8 T cells; higher lymphoid infiltrates in responders to both therapies and a more clonal and diverse T cell infiltrate in responders to nivolumab monotherapy were also observed.

The open-label OpACIN-neo trial was designed to identify an effective but more tolerable dosing schedule of ipilimumab plus nivolumab, with patients randomized to one of three different ipilimumab plus nivolumab dosing regimens [39]. Of these, two cycles of ipilimumab 1 mg/kg plus nivolumab 3 mg/kg every 3 weeks was identified as tolerable and effective, inducing a pathological response in 77% of patients. At 2-year follow-up, almost all high-grade adverse events had resolved to ≤ grade 1, except for grade 2 endocrinopathies. More than 80% of patients were relapse-free at 2-years without adjuvant treatment, and RFS remained significantly higher for patients with pathologic response versus non-responders (97% versus 36%). High baseline TMB and IFN-γ signature expression were associated with response outcomes [40]. This is being further investigated in the DONIMI study, which will assess nivolumab alone, with a class 1 histone deacetylase inhibitor, domatinostat, or with both domatinostat and ipilimumab, in 40 patients with newly diagnosed or recurrent disease [41]. In patients with IFN-γ signature-high melanoma, radiological and pathological response rates were robust with nivolumab alone or combined with domatinostat, whereas in IFN-γ signature-low patients, response rates were low with nivolumab plus domatinostat and with nivolumab plus domatinostat plus ipilimumab.

In another recent study, 30 patients with clinical stage III or oligometastatic stage IV melanoma with Response Evaluation Criteria in Solid Tumors (RECIST) v1.1 disease were treated with neoadjuvant followed by adjuvant nivolumab plus the anti-LAG3 antibody relatlimab [42]. The pCR rate was 59% and MPR rate was 66%. One-year RFS was 100% for patients with MPR compared to 80% for non-MPR patients. Nivolumab plus relatlimab was well tolerated, with no treatment-related grade ≥ 3 or higher toxicities noted in the neoadjuvant setting and no surgical delays due to treatment-related toxicity. In the adjuvant setting, 26% of patients had grade 3 toxicity, with adrenal insufficiency the most frequent. Increased effector CD8 + T cells and decreased immunosuppressive M2 macrophages correlated with a favorable response.

**Fig. 2:**
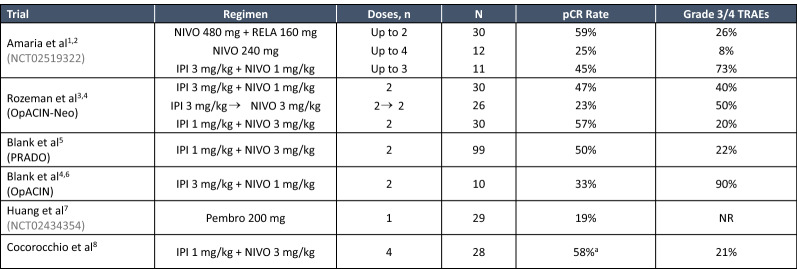
Overview of Neoadjuvant Immunotherapy Trials in Melanoma

Overall, the ipilimumab 1 mg/kg plus nivolumab 3 mg/kg appear to be the most robust regimen, although nivolumab plus relatlimab appears to be comparable. Ongoing questions include whether adjuvant therapy is required after neoadjuvant treatment, especially in patients with robust pathological responses. We also need to know if neoadjuvant therapy can reduce the extent of surgery and whether baseline tumor features can be used to help to delineate which neoadjuvant treatment regimen might be most appropriate (Fig. 2).

## Non-melanoma skin cancer

Similar to melanoma, non-melanoma skin cancer (NMSC) including cutaneous squamous cell carcinoma (CSCC) has a high mutational burden, providing a strong rationale for the use of immunotherapy. Several trials have shown the efficacy of PD-1 inhibition in CSCC [43–46]. However, earlier application may potentially improve outcomes still further.

In an MD Anderson Cancer Center trial, 20 patients with primary or recurrent stage III-IVA CSCC of the head and neck (measurable disease of > 1.5 cm per RECIST 1.1) with planned curative-intent surgery and adjuvant radiation received two cycles of neoadjuvant PD-1 inhibition with cemiplimab, [47]. The primary endpoint was ORR per RECIST 1.1. Secondary endpoints included pCR, MPR, disease-specific survival (DSS), disease-free survival (DFS), and OS. Safety was assessed and biomarkers of response were evaluated.

Although only six patients (30%) had partial imaging responses by RECIST, 15 patients (75%) had either a pCR (11, 55%) or a MPR (4, 20%). This illustrates the underestimation of pathological response by radiographic imaging. Of the 15 pathologic responders, 12 (80%) did not receive adjuvant radiation as planned and at a median follow up of 22.6 months all remain free of disease. An additional 2 (10%) patients had a pPR, were treated with adjuvant therapy and also remain disease free. Three (15%) patients had > 50% viable tumor remaining at the time of surgery and were classified as non-responders. These patients had neither a clinical nor pathologic response, and all recurred. In retrospect, 2 of these patients had borderline resectable disease at presentation, underscoring the importance of careful patient selection. Histopathologic assessments showed significant heterogeneity, even among responders, including in the extent of inflammatory infiltrate. The 12-month DSS, DFS, and overall survival (OS) rates were 95%, 90%, and 95%, respectively.

Pre-treatment gene expression profiling revealed an inflamed TME in patients with pCR or MPR, indicating that a favorable immune microenvironment prior to cemiplimab treatment is associated with pathologic response. Mass cytometry CyTOF analysis of pre- and post-treatment tumor samples showed changes in the tumor immune microenvironment after cemiplimab treatment were also associated with pathologic responses, in particular enrichment of memory CD8 + T-cell clusters. A regulatory T cell subset (CD3 + CD4 + FOXP3 +) and a myeloid cell subset expressing inhibitory marker VISTA (CD68 + CD14 + VISTA +) were associated with non-response and may help explain why some patients failed treatment. A confirmatory multicenter phase II trial with 76 patients receiving neoadjuvant cemiplimab followed by surgery and adjuvant cemiplimab, radiation or observation was recently completed (clinicaltrials.gov: NCT04154943).

**Fig. 3:**
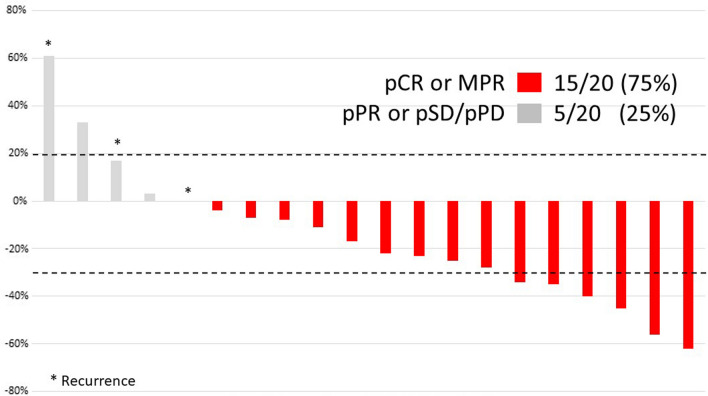
Immunotherapy CSCC-Pathology Results. Three patients who recurred had neither clinical or pathologic response. Two patients who recurred had borderline resectable disease underscoring the importance of patient selection

In conclusion, NMSC, in particular CSCC, is highly responsive to immunotherapy and the earlier application of immunotherapy may improve outcomes. Neoadjuvant immunotherapy is well tolerated and demonstrates durable responses in CSCC with no surgical delays and diminished need for adjuvant radiation therapy. Careful patient selection for neoadjuvant immunotherapy remains paramount, and a favorable baseline TME may predict response (Fig. 3).

## Refining pathological response assessment in neoadjuvant therapy for melanoma

Pathological assessment is critical for evaluating neoadjuvant therapy and is typically the primary endpoint in clinical trials. It is therefore important that it is performed in a standardized manner; the INMC has developed guidelines for pathological evaluation of specimens to facilitate this. Tumor size assessed by radiology correlates with the tumor bed size but not the extent of residual viable tumor [48].

Reproducibility of pathological response is very important. In the OpACIN-neo trial, patients were recruited in Australia and Europe (the Netherlands and Sweden). Pathological responses were evaluated at the respective centers at which patients were recruited. Lymph node dissection specimens of 83 patients were shared across centers and re-evaluated by pathologists at the other sites, with excellent interobserver reproducibility for pathological response category and percentage pathological response being observed [48]. Standardization of pathological assessment has also enabled data to be pooled from different melanoma neoadjuvant clinical trials in order to compare efficacy of treatments [4].

Pathological response is an excellent predictor of clinical outcome in melanoma patients treated with neoadjuvant therapies. As a consequence, pathological response evaluation enables clinicians to provide information to patients on their long-term prognosis after a short period of neoadjuvant therapy and may also inform clinical decision-making. In the PRADO trial of combination neoadjuvant nivolumab and ipilimumab, patients with a pCR or near pCR in the index node were followed-up with observation only, whereas patients with a partial or no response underwent therapeutic lymph node dissection (TLND); patients with no response in their TLND then received adjuvant nivolumab or dabrafenib plus trametinib while partial response patients underwent observation [49]. Thus, pathological response is being used to personalize treatment and de-escalate surgery. At a minimum of 12 weeks follow-up, 70 of 99 patients (71%) had a pathological response in the index lymph node and 60 patients (61%) had MPR in the index lymph node and did not undergo therapeutic lymph node dissection. These patients had fewer surgery-related adverse events and much higher quality-of-life scores.

In OpACIN-neo, almost all patients with recurrence had ≥ 70% residual viable melanoma. Type of pathological response also predicted outcome, with patients with a high fibrosis subtype of treatment response having the strongest association with lack of recurrence and prolonged RFS. These data are being used to develop a predictive score for relapse [48]. Patients with a pCR or near pCR had a high degree of hyaline fibrosis. However, pigment-laden macrophages, necrosis and tumor-infiltrating T cell infiltrate did not correlate with category of pathological response. Even in patients with no pathological response, hyaline fibrosis is associated with prolonged RFS. Similarly, patients treated with neoadjuvant dabrafenib and trametinib who achieved pCR had longer RFS. Hyalinized fibrosis was again associated with longer RFS in patients whereas necrosis and/or immature/proliferative fibrosis correlated with shorter RFS [50]. Patients with high fibrosis have differences in circulating T, natural killer (NK) and myeloid cells in peripheral blood compared with low fibrosis patients. Subtype of pathological response following neoadjuvant therapy appears to be important in determining outcomes. High TMB and high IFN-γ signature score are also associated with pathologic response and low risk of relapse [48]. In addition, gastrointestinal microbial diversity is low in both non-responders and patients with severe immune-related adverse events.

In conclusion, pathological response assessment is reproducible. Radiology tumor size correlates with the tumor bed size but not pathological response. Pathological, peripheral blood, molecular and microbiome-related factors may all be associated with response and prolonged survival.

## Conclusions

Administration of immunotherapy in a neoadjuvant setting offers the potential to help improve outcomes for patients with high-risk, surgically resectable cancers. Neoadjuvant immunotherapy may improve operability, prime the antitumor immune response at an early stage, minimise micrometastatic disease, and prevent tumor relapse. Additionally, neoadjuvant treatment also provides an opportunity for early assessment of tumor response to therapy using surrogate endpoints of clinical efficacy at surgery. Neoadjuvant immunotherapy rather than upfront surgery and adjuvant therapy has the potential to become a new standard of care in several settings.

The Neoadjuvant Immunotherapy Across Cancers at the 2021 Immunotherapy Bridges meeting provided an invaluable opportunity to bring together experts in immunotherapy and neoadjuvant treatment. Highlighted in this session were changes to standard of care as well as ongoing trials which continue to evaluate neoadjuvant immunotherapies in different tumors. This session also highlighted the neoadjuvant platform as a unique, pan-cancer opportunity to leverage biomarker analyses from robust tissue collections to gain novel insights into mechanisms of response and resistance to therapies, potentially informing subsequent development of immunotherapy in other indications. While surrogate endpoint validation for chemotherapy is well established, pathologic response to neoadjuvant immunotherapy is in development and will be critical to placing biomarker insights, clinical response, and outcomes into context. Treatment of surgically resectable cancers with neoadjuvant immunotherapy remains of significant interest to improve outcomes for patients across cancer types and stages.

## Data Availability

Not applicable.

## Abbreviations

CRC: Colorectal cancer
CSCC: Cutaneous squamous cell carcinoma
CT: Computed tomography
CTLA-4: Cytotoxic T-lymphocyte-associated antigen-4
dMMR: Mismatch-repair-deficient
DFS: Disease-free survival
DSS: Disease-specific survival
EFS: Event-free survival
HR: Hazard ratio
ICOS: Inducible T-cell costimulator
IFN: Interferon
INMC: International Neoadjuvant Melanoma Consortium
MPR: Major pathological response
MSI: Microsatellite-instability-high
NIF: Nodal immune flare
NK: Natural killer
NMSC: N-melanoma skin cancer
NSCLC: Non-small-cell lung cancer
ORR: Objective response rate
OS: Overall survival
pMMR: Mismatch-repair-proficient
pCR: Pathological complete response
PD: Programmed death
PD-L1: Programmed death ligand
RECIST: Response evaluation criteria in solid tumors
RFS: Recurrence-free survival
TLND: Therapeutic lymph node dissection
TMB: Tumor mutational burden
TME: Tumor microenvironment
TNBC: Triple-negative breast cancer
VISTA: V-domain Ig suppressor of T cell activation
